# Teachers’ Perspective on Strategies to Reduce Sedentary Behavior in Educational Institutions

**DOI:** 10.3390/ijerph17228407

**Published:** 2020-11-13

**Authors:** Nastja Podrekar, Kaja Kastelic, Nejc Šarabon

**Affiliations:** 1 Human Health in the Built Environment, InnoRenew CoE, 6310 Izola, Slovenia; nastja.podrekar@innorenew.eu (N.P.); kaja.kastelic@innorenew.eu (K.K.); 2 Faculty of Health Sciences, University of Primorska, 6310 Izola, Slovenia; 3 Andrej Marusic Institute, University of Primorska, 6000 Koper, Slovenia

**Keywords:** student-furniture mismatch, school furniture, classroom physical activity break, sit-to-stand desks, sedentarism

## Abstract

Standing desks and physical activity breaks can counteract the negative consequences of sedentarism at school. However, the implementation of these strategies should not restrict the pedagogical process. The aim of this study was to assess teachers’ perspectives on strategies to reduce sedentary behavior (SB) of students in the classroom. An online survey was conducted, and the answers were analyzed using descriptive statistics and frequency distribution. The relationships between the variables were assessed using Spearman’s coefficient and a chi-square test. Most teachers believed that a student–furniture mismatch was present. The most common reasons given for not using standing desks were concerns about desks being uncommon and their potential to disrupt the class. However, the majority of the teachers believed it feasible to perform physical activity (PA) breaks during classes. Further intervention studies are needed to determine for which courses the use of standing desks are feasible, for what time periods standing desks should be used, and the appropriate number and layout of standing desks in classrooms.

## 1. Introduction

The first environment in which an individual is exposed to prolonged sitting is the school environment. In 1981, Mandal [1] renamed modern humans “homo sedens” due to the many hours spent in a sitting position from childhood onward. In school, students learn and acquire habits they will maintain throughout their lives. Therefore, students should be educated about the negative consequences of prolonged sitting and sedentary behavior (SB), such as unfavorable body composition, decreased fitness, lowered scores for self-esteem, and decreased academic achievement [2], and should learn strategies to counteract these negative effects.

Behaviors can be affected by space and elements in a building [3]. In this context, interior space has the potential to promote the physical activity (PA) of a user [4]. Specifically, the designs of school buildings and grounds, including form, layout, and elements of a building, may enhance children’s PA levels, especially when combined with social and educational support [5]. Within the school setting, PA can be promoted during regular classes, PA classes, recess, and break time [6]. The longest periods of uninterrupted sitting are anticipated to occur during classes, and interventions to reduce SB in the classroom are therefore of great importance.

Use of standing desks, which allow students to stand during lessons, may decrease classroom sitting time by 52 min per day on average [7]. One study reported an increase in moderate-to-vigorous physical activity (MVPA) of 20 min per day after standing desks were implemented [8]. Reallocating time spent engaged in SB to PA is important in enhancing the cardio-metabolic health of children [9]. Substituting one hour of SB with MVPA was associated with 6.1% (95% CI: 2.1, 9.2) lower visceral adipose tissue [10] and improved insulin sensitivity [9]. In addition, using standing desks can increase students’ energy expenditures in the classroom by up to 25% [11,12], thus affecting the energy balance that is critical to changing the trajectory of obesity in youth. Using standing desks is also associated with lower musculoskeletal discomfort, especially in the neck, shoulders, and lower back (OR 0.52–0.74), when compared to a sitting-only condition [13]. Additionally, children agreed that it is easier to work using a standing desk, and evaluated standing desks as a positive change in the classroom environment [8].

The dimensional suitability of furniture is important in ensuring proper posture and preventing musculoskeletal discomfort. The rate of childhood growth varies among students [14], and the anthropometric dimensions of students of the same age differ. However, school furniture in the classroom is the same for all students, regardless of their size. Research has shown that a mismatch between students and furniture can increase the risk of developing lower back pain [15,16] due to increased strain on the musculoskeletal system [17]. Numerous studies have observed the mismatch between the dimensions of students’ bodies and furniture in various countries [18,19,20,21], with a mismatch occurring in 60–99% of cases for seat height, 55–99% for seat depth, and 52–99% for desk height [20,21,22,23,24,25,26].

Performing classroom physical activity breaks (CPABs) might also influence children’s activity levels in school. An increase of six minutes of MVPA during class was observed among children performing CPABs [8]. Children performing CPABs in the classroom were more likely to reach 30 min of MVPA when in school compared to children not performing activity breaks (OR 1.75, 95% CI = 1.22, 2.25, *p* = 0.002) [27]. In addition, CPABs may improve children’s behavior [22,23,24]. Children believed that CPAB positively influenced their health and learning capabilities [28].

Despite the positive effects of interventions to reduce SB in schools, it is important to consider the feasibility of introducing new approaches in the school environment, as the proposed solutions should not restrict the learning process. It is also crucial that teachers be prepared to accept the proposed changes in the school environment, as they are the ones who encourage and motivate students to change existing habits or introduce new ones. It has been indicated that the use of standing desks during lessons depends to a large extent on the support of teachers and parents [29].

Based on the recognized gaps in knowledge, the first nationwide survey for primary, secondary, and university teachers in Slovenia was conducted. The aim of this study was to assess teachers’ opinions on (1) the suitability of current school furniture in relation to body dimensions of students, (2) using standing desks in classrooms, (3) performing CPABs, (4) the postures of students in class, and (5) purchasing new school furniture.

## 2. Materials and Methods

### 2.1. Participants

The study population consisted of a convenience sample of teachers and professors from Slovenian primary and secondary schools as well as universities. Only certified teachers were eligible to participate in the survey. Administrators and other school personnel did not participate in the study.

About 1200 teachers were invited to complete the survey. A total of 234 teachers (19.5% response rate) replied to the survey and 170 teachers (72.6%) completed the survey in full. Our sample consisted of 122 teachers from primary schools (age 47.60 ± 10.25), 31 teachers from secondary schools (age 53.06 ± 7.85), and 17 professors from universities (age 40.00 ± 10.08). On average, teachers had 19.7 ± 12.6 years of teaching experience. Detailed characteristics of the teachers are shown in Table 1.

### 2.2. Survey Instrument

The survey instrument was developed to assess the perceived roles, feasibility, and barriers of teachers regarding the suitability of current school furniture, standing desks, and implementation of PA breaks in the classroom. The survey was conducted in Slovenia. Before the administration of the survey, a sample of teachers (N = 5) reviewed the survey and assessed its clarity and relevance to the research question. After the review, two questions were removed, and five questions were adjusted. Then, pilot tests were conducted by teachers (N = 8) to test the test–retest reliability. Teachers completed the survey at baseline and one week later. The weighted Cohen’s Kappa with equal spacing and Spearman’s rho correlation for questions on an ordinal scale are presented in Table 2.

The final version of the questionnaire contained 35 questions: 11 open questions, 15 closed questions, and nine rating scale questions. Items in the rating scale questions were rated on a nine-level scale, with responses ranging from 1 = not at all/absolutely disagree to 9 = considerably/absolutely agree. The final version of the questionnaire is presented in Table S1, Supplementary Materials.

### 2.3. Procedure

The online survey was distributed to the schools by the Association of Principals of Slovenia and, additionally, to two out of the three public universities in Slovenia. The survey was available from 28 November 2019 until 4 February 2020. The survey was administered with 1 ka Anketa, a secure online survey platform widely used in Slovenia. Principles were informed about the purpose of the survey and encouraged to forward the survey link to their teachers. Contact information (name, last name, and e-mail) of the researcher was provided in the introduction of the questionnaire so that the participants could contact the researchers and ask additional questions. Participation in the study was voluntary and teachers received no compensation for completing the survey. Responses were anonymous, and teachers could skip any question in the survey and stop the survey at any time. Study procedures were approved by the Slovenian National Medical Ethics Committee number 0120-557/2017/4.

### 2.4. Data Analysis

The data were analyzed using Microsoft Excel (version 2002, Microsoft, Washington, DC, USA) and Statistical Package for the Social Sciences (version 26.0, 2019, IBM Corp., Armonk, NY, USA). Descriptive statistics and frequency distributions were calculated, and the relationship between variables was assessed using Spearman’s coefficient (0 = no correlation, 0.1–0.2 = weak correlation, 0.3–0.5 = moderate correlation, 0.6–0.7 = strong correlation, 0.8–0.9 = very strong correlation, 1 = total correlation) [30] and a chi-square test. Bonferroni correction was used for the post-hoc tests. The statistically significant differences were accepted at the significance level α < 0.05. Representative teacher comments were selected from the open questions.

## 3. Results

### 3.1. Suitability of Classrooms and School Furniture

**Figure 1:**
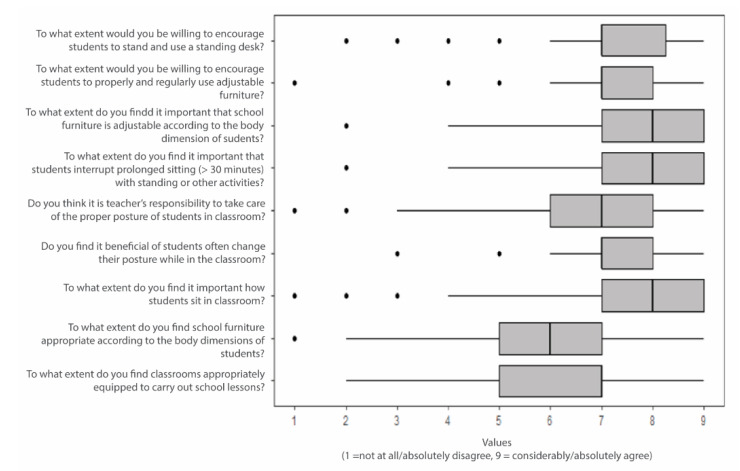
Teachers’ responses from the survey. The box presents the interquartile range (IQR), from the 25th (Q1) and 75th (Q3) percentile. The horizontal line presents the minimum (Q1 − 1.5*IQR) and maximum (Q3 + 1.5*IQR) value in the data. Bold lines represent the median values, and points present potential outliers.

Most teachers (85.7%) found the current school furniture to be unsuitable for students. Teachers most frequently mentioned the mismatch between the body dimensions of students and the dimensions of the furniture. They emphasized that the furniture was too small, especially for tall boys, and computer screens were positioned too low. On the other hand, teachers noted that the seats were too high for smaller students, so they could not reach the floor with their feet. They noted that the furniture was not suitable for overweight students, and there were significant anthropometric differences between boys and girls. In addition to chairs, they also mentioned that desks were often too narrow and too short. In general, the teachers argued that school furniture was old and outdated. About two thirds of teachers (68.0%) thought that the classrooms were not suitably designed and equipped for teaching. They also pointed out that the classrooms were too small, and blackboards and shelves were often placed too high, making it difficult for students to reach them. The teachers mentioned that the chairs were uncomfortable to sit on and the white color of the furniture led to glare, making the eyes tired. These discrepancies were mentioned by teachers in primary and secondary schools. The teachers’ answers are shown in Figure 1.

Question: Where do you see the biggest mismatch between school furniture and students?

Teachers’ comments:

» Chairs and desks are too high for the students (unfortunately, manufacturers sell school desks and chairs based on the age of the students, but this [in my opinion] is often not appropriate). Then the furniture is ordered in a hurry and it is not suitable for students. «

(Primary School teacher, age 34)

» Every day in my classroom, there are students from the 1st to the 4th grade sitting, and they all use the same desks and chairs. Also, the height of the blackboard is not at the height of the eyes of the children but at the height of the eyes of the adults. «

(Primary School teacher, age 27)

» We make every effort (with the support of the principal) to ensure that chairs and desks are the right height for each student. In my opinion, some of the cupboards intended for teachers take up too much space. In other words, the classrooms are too small. «

(Primary School teacher, age 42)

» Some students have different motor skills and would need custom-design furniture. «

(Primary School teacher, age 42)

» Desks and chairs are the same for students aged 10 to 14 or 15, but during this time, the children grow taller by half a metre or more. «

(Primary School teacher, age 30)

### 3.2. Use of Standing Desks in the Classroom

The majority of teachers (88.4%) consider it feasible to interrupt longer sitting periods in classes (more than 30 min) by standing. More than 90% of teachers encourage students to stand up during breaks. The most common reasons given for not using strategies to interrupt sitting were the following: it is not common, it disrupts the class, and it changes the organization of work. The teachers mentioned that use of a standing desk was not feasible during all school subjects (e.g., mathematics). More than half of the teachers (55.2%) acknowledged carrying out activities to interrupt sitting in class (e.g., going to the blackboard), while less than 10% of the teachers claimed to never interrupt sitting during lessons. Teachers who found interrupting long-term sitting important were more likely to be prepared to encourage students to use standing desks during class (r = 0.50, *p* < 0.01).

Question: Why do you not interrupt sitting during lessons with other activities, such as standing?

Teachers’ comments:

» I never really thought about that. Maybe then there would not be enough time to go through the topic. «

(Secondary School teacher, age 45)

» Since this is not done habitually, it would lead to confusion in the classroom, pupils would not be able to focus, and the teaching message, the purpose of it, would be misunderstood. «

(Secondary School teacher, age 54)

Four teachers (2.4%) replied that they had adjustable furniture in at least one classroom, and 33 teachers (19.4%) said they had standing desks in at least one classroom. Teachers who already used standing desks in the classroom stated that using standing desks for 40 min a day was feasible. In contrast, some teachers (7.5%) thought that using standing desks was not feasible, mainly due to the expected confusion and a lack of cooperation from students. They suspected that standing desks would disturb the teaching process, as it might increase the interaction between students and divert attention from the subject matter. Teachers emphasized that support from the principal was crucial in order to use standing desks in the classroom.

Question: Why do you think that the use of standing desks in the classroom is not feasible?

Teachers’ comments:

» It is not necessary. They sit for 45 min or less and then have a break in which they can move. «

(Primary School teacher, age 44)

» Moving around in the middle of the class does not take a moment, but precious minutes. The curriculum is overfilled, not everything is done and then there are various things that interrupt the teaching process. In the end, this is another thing that would disrupt the teaching process. «

(Primary School teacher, age 38)

### 3.3. Performing Physical Activity Breaks during Class

Almost all teachers (95.3%) believed that taking CPABs during lessons was feasible, which was supported by the fact that half of the respondents (51.2%) already took PA breaks. Teachers who did not consider PA breaks to be feasible argued that this would disrupt the pedagogical process. Some teachers believed that the introduction of CPABs should start in the first grade. PA breaks were most often implemented by primary school teachers (*p* < 0.01).

Question: Why do you think that performing physical activity breaks is not feasible during class?

Teachers’ comments:

» I think it would be very difficult to get the students to exercise. We should start in the first grade. «

(Secondary School teacher, age 38)

» It seems strange to perform this with adults (students at the faculty)–probably simply because this is not (yet) common practice. «

(University Assistant, age 29)

### 3.4. Body Posture When Sitting in Class

Almost half of the teachers (48.0%) stated they reminded the students to sit correctly every day. On the other hand, 10.7% of the teachers did not pay attention to students’ postures. From a selection of postures (presented in Table S1, Supplementary Materials), more than half of the teachers (68.6%) chose image “D” as an example of the most appropriate posture when sitting. About one fifth of teachers (21.7%) chose image “B” and less than one-tenth (9.7%) chose image “C”.

Teachers argued that suitable school furniture enhances proper posture during sitting and promotes well-being, thus promoting the attention and focus of the students as well. The teachers also pointed out that lessons on the ergonomics of sitting should begin in the first grade.

Question: Why do you think it is important how students sit in class?

Teachers’ comments:

» Unfortunately, more and more often students sit in awkward postures, but there is not much we as teachers can do about it, as we can hardly say anything to remind them. «

(Primary School teacher, age 58)

» The educational system is a very big factor in our sedentary lifestyle. So many hours spent in a sitting position behind ‘strange’ desks has consequences. And if the students have been sitting for a long time, they should at least sit ‘properly’. «

(University Professor, age 45)

» Yes, forced posture reduces creativity. But it is not necessary for them to sit. They can stand, squat, lie... the important thing is that they work. «

(Primary School teacher, age 62)

» Because I think the way they sit has a significant impact on the development of their spine and possible deformations, I believe that the students must sit comfortably (with their feet on the floor and their hands at the appropriate height) because only then can they concentrate on their work. Otherwise, sitting itself is an interruption. «

(Primary School teacher, age 38)

» Because of physical development, and because it easier to learn to sit properly at younger age. «

(Primary School teacher, age 27)

### 3.5. Teacher Participation in the Purchase of School Furniture

About two thirds of the teachers stated that they (1) never discuss the topic of school furniture at conferences and meetings (64.0%) and (2) have never been involved in the purchase of new school furniture (66.3%). The majority of teachers (67.1%) were interested in school ergonomics and would be willing to attend a seminar or training on this topic.

## 4. Discussion

For the long-term implementation of strategies to counteract the negative effects of SB in educational institutions, it is important to consider the views, barriers, and perspectives of teachers. Results of this study showed that most (but not all) teachers believed the use of standing desks in schools is feasible. The teachers considered CPABs to be possible to implement; in fact, more than half of the teachers already include PA breaks in their lessons.

In this study, more than half of teachers believed that classrooms were not currently suitable for teaching, that the furniture was old and outdated, and that school furniture dimensions did not match the body dimensions of students. The latter belief is supported with the student-furniture mismatch that has been objectively measured and calculated by several studies around the world [20,21,22,23,24,25,26]. However, these studies have focused mostly on school furniture, and knowledge of the suitability of schools as a whole is lacking. The occurrence of adjustable furniture and standing desks in Slovenian schools varied. About 20% of the teachers reported having standing desks in at least one classroom, but since the survey was conducted anonymously, we cannot say whether these teachers came from the same school. At present, standing desks are mainly used for students with special needs. Slovenian teachers who already use standing desks in the classroom stated that it is feasible to use standing desks for 40 min a day. Accordingly, studies have shown that standing desks can be used between 20 and 60 min per school day [8,31]. Slovenian teachers stated that the use of standing desks is not suitable for all school subjects, especially mathematics. This is in line with a Japanese study that reported standing desks were most often used in social science subjects such as sociology, ethics, and arts, but only once in mathematics [8]. It has been shown that standing desks are appropriate for group work [8], while the optimal number of standing desks and the furniture layout in classrooms is still unknown and varies among studies [32,33,34].

In the survey, some teachers stated that the use of standing desks could lead to increased interactions between students and, consequently, could interrupt the studying process. Based on the opinion of Belgian teachers, disruption in the classroom when using standing desks was considered a risk mainly among older teachers who are more used to the traditional learning process at school [32]. Despite some negative opinions regarding standing desks, studies show that standing desks have been well accepted by teachers [32,35,36,37]. Standing desks have also been positively accepted by Portuguese teachers, as only one in nine teachers did not want to use standing desks after the desks were used in classrooms [34].

Almost all our participants stated that it is feasible to perform CPABs in the classroom. This is important since it has been shown that the participation and involvement of students in performing activity breaks in schools is strongly dependent on the support of teachers [38]. According to a review, students show better classroom behavior after 10 min of MVPA breaks or a shorter and more intensive five-minute break [39]. The Slovenian teachers suggested starting CPABs in the first grade, which is important to consider because behavior patterns acquired in early childhood can be maintained throughout adult life. Activity breaks are most often performed by primary school teachers, which can be explained by the opinion of a university assistant who stated that they were uncomfortable exercising with university students because they were already adults. Additional general strategies and guidelines to support schools and teachers are needed in order to consistently implement PA breaks at school [40].

Given the key role of teachers in fostering the adoption of new habits among students, it is important that teachers be educated about the negative consequences of sitting. Although there has been no final consensus on what the best sitting posture is [41], more than half of the teachers selected a photo that matched the most natural shape of the spine without an excessive muscle tone. Previous research has shown that upright sitting with lower extremities bent 90° might not be the optimal posture for long-term sitting [1,42]. Mandal [1] suggested a higher school chair with a forward inclination of the seat pan to better accommodate children when they perform school-related tasks such as reading and writing. Indeed, a more neutral body posture of students was observed when using the school furniture proposed by Mandal [43,44]. However, in Slovenian schools, standard school chairs are designed with a flat seat pan. Therefore, the photos presented in the Figure 1 present the body postures most plausible and typical of an individual seated on a standard chair in a Slovenian school.

It is encouraging that two-thirds of Slovenian teachers find topics related to school ergonomics interesting and would be willing to participate in a knowledge-building seminar. This is crucial, as teachers trained in ergonomics could have a greater impact beyond just the ergonomic furniture itself [45]. It is also important that teachers be involved in the purchase of new school furniture, as they are the ones who use it every day. The effort and willingness of teachers are crucial in encouraging students to participate and adopt strategies to fight SB in the classroom and ensure their long-term implementation.

## 5. Limitations

The completed study has certain limitations, such as low teacher response (19.5%), and a possible response only from those teachers who find this topic interesting and important. The opinions of the teachers collected in this study cannot be generalized due to the small sample size and demographic imbalances: the sample was made up of more than 80% women, more than 80% teachers were older than 45 years, and 70% of participants were primary school teachers. A detailed statistical comparison of results between the primary and secondary teachers and university professors was not possible due to the sample size differences in groups. Additionally, the online survey method could have had certain weaknesses: participants might (1) not have understood the questions correctly, (2) have found the approach too impersonal to be fully engaged, or (3) have lacked the technical expertise to provide precise responses. Therefore, the results should be interpreted considering the presented limitations.

## 6. Conclusions

In this study, an online survey was conducted to assess the opinions of teachers regarding strategies to reduce SB of students in schools. The results of our study show that most teachers believe that there is a mismatch between the dimensions of students’ bodies and furniture dimensions, and that current furniture is outdated. The teachers considered CPABs to be feasible to perform, which was confirmed by the fact that more than half of the teachers already implemented PA breaks during lessons. Almost half of the teachers corrected the students’ postures every day and believed that it is a teacher’s duty to ensure that students’ postures are correct. Although most teachers believed that the use of standing desks in schools was feasible, they pointed out that standing desks were not suitable for all school subjects.

Changing classroom environments in favor of reducing classroom SB has the potential to promote students’ well-being without disrupting the pedagogical process. Based on the results of this study, the teachers were aware of the importance of the strategies to promote the health of students in the classroom, and certain implications could be pointed out:

- reorganization and redesign of the current school environment and school furniture in Slovenia is desired;
- regular anthropometry measurements should be incorporated to ensure that students are using suitable furniture that promotes good posture when sitting;
- standing desks in the classroom could be used for at least 40 min daily, although not for all school subjects;
- the implementation of strategies to reduce SB should begin in the first grade, so that teachers and students accept these interventions as a normal part of the learning process;
- for the implementation of new strategies to enhance the well-being of students in the school environment, support of the principal that is in compliance with standards and school-related restrictions is important;
- additional general strategies and guidelines are needed to support schools and teachers to consistently implement PA breaks at school;
- along with the suggested strategies, budgetary and other constraints that influence decision making by school personnel should be considered; and
- regular seminars related to school ergonomics and SB for teachers should be organized.

Further intervention studies are needed to determine for which courses the use of standing desks are feasible, for what time periods standing desks should be used, and what the appropriate number and layout of standing desks in a classroom should be. Similarly, for CPABs, suitable frequencies and types of exercise should be researched and introduced to principals and teachers to encourage regular PA breaks during class.

## Figures and Tables

**Table 1:** Characteristics of the participants.

	Primary School Teachers (N = 122)		Secondary School Teachers (N = 31)		University Professors (N = 17)	
	N	%	N	%	N	%
Females	111	90.98%	23	74.19%	8	52.94%
Males	11	9.08%	8	25.81%	9	47.06
Participants older than 45 years	77	63.11%	29	93.55%	4	23.53

**Table 2:** Reliability of the survey questions.

	Weighted Kappa	95% Confidence Interval	Spearman’s Rho	*p* Value
Question 1	0.36	0.11 to 0.60	0.78	0.02
Question 2	0.36	0.11 to 0.60	0.93	<0.01
Question 3	0.77	0.55 to 0.98	0.89	0.02
Question 4	0.76	0.41 to 1.00	0.41	0.30
Question 5	0.55	0.23 to 0.88	0.67	0.07
Question 6	0.55	0.23 to 0.88	0.68	0.06
Question 7	0.92	0.77 to 1.00	0.84	0.01
Question 8	0.74	0.39 to 1.00	0.88	0.01
Question 9	0.77	0.23 to 1.00	0.90	0.01
Question 10	0.74	0.44 to 1.00	0.91	0.01

## Supplementary Materials

The following are available online at https://www.mdpi.com/1660-4601/17/22/8407/s1, Table S1: The Questionnaire.
