# Supplementary material for: Teachers’ Perspective on Strategies to Reduce Sedentary Behavior in Educational Institutions
Source: Int J Environ Res Public Health. 2020 Nov 13;17(22):8407. doi: 10.3390/ijerph17228407 (PMC7696757; doi:10.3390/ijerph17228407)
Supplement: Supplementary file 1 [file ijerph-17-08407-s001.pdf]

SUPPLEMENTARY MATERIALS

The Questionnaire

V1 - To what extent do you find the classrooms to be suitably equipped for class?

Mark with the slider on the bottom line.

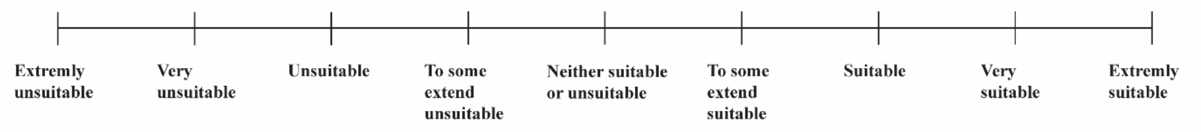

V2 - To what extent do you find school furniture appropriate for the body dimensions of students??

Mark with the slider on the bottom line.

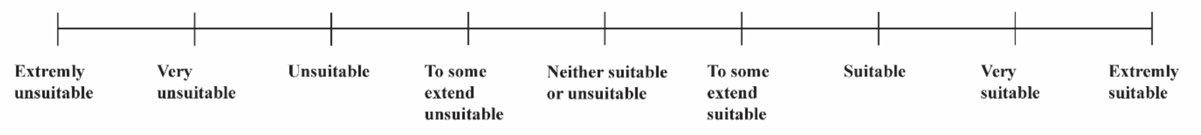

V3 - Where do you see the biggest discrepancies between school furniture and the body dimensions of students?

V4 - To what extent do you find it important how students sit during class?

Mark with the slider on the bottom line.

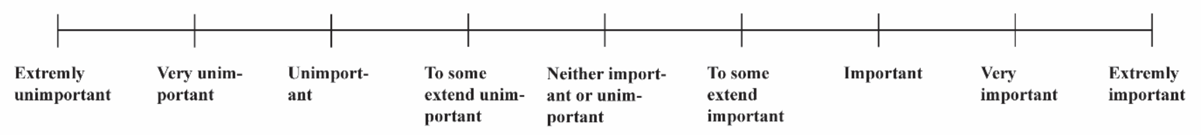

V5 - Why do you find it important how students sit during class??

V6 - Choose a picture that you think shows the most correct posture while sitting in class.

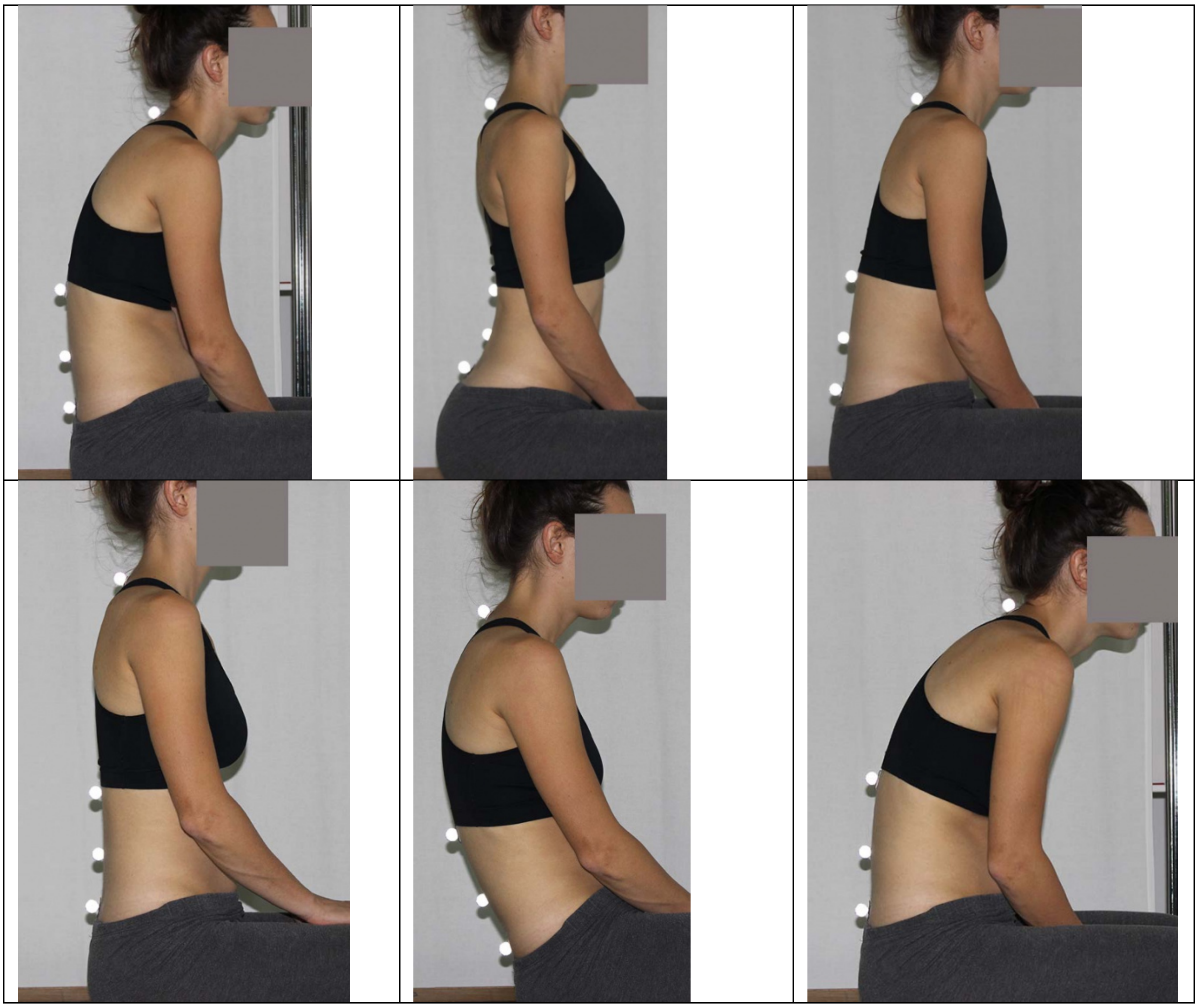

V7 – How often do you remind students about posture while sitting in class??

- ☐ Every day
- ☐ At least once a week
- ☐ At least once a month
- ☐ 1-2-times a year
- ☐ Never

V8 - Do you think it is useful if students often change their posture while sitting in class?

Mark with the slider on the bottom line.

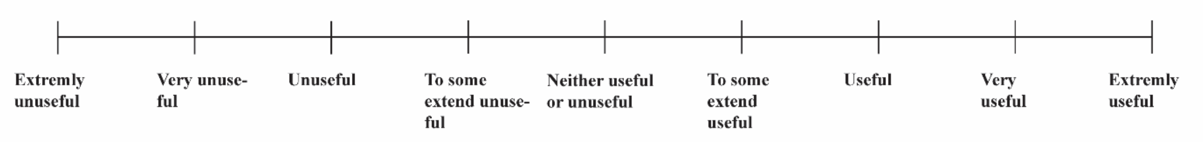

V9 - Do you think that it is the teacher's duty to take care of the proper posture of students during class?

Mark with the slider on the bottom line.

Extremly disagree	Very disagree	Disagree	To some extend disagree	Neither agree or disagree	To some extend agree	Agree	Very agree	Extremly agree

V9_1 - Would you attend a seminar on sitting ergonomics, the importance of proper posture while sitting in class?

- ◯ Yes
- ◯ No

V10 - How often do you interrupt sitting during class with other activities (e.g. standing)?

.

- ☐ Every day
- ☐ At least once a week
- ☐ At least once a month
- ☐ 1-2-times a year
- ☐ Never

V11 - Why don’t you interrupt sitting during class with other activities (e.g. standing)?

Example: because of the confusion, because of the organization of work, because it is not usual.

V12 - To what extent do you find it important for students to interrupt prolonged sitting (more than 30 minutes) with standing or other activities?

Mark with the slider on the bottom line.

Extremly unimportant	Very unim-portant	Unimport-ant	To some extend unim-portant	Neither import-ant or unim-portant	To some extend important	Important	Very important	Extremly important

V13 - Do you have school chairs and desks in classrooms that allow adjustment according to the body dimensions of students?

- ☐ Yes, chairs and desks
- ☐ Yes, chairs
- ☐ Yes, desks
- ☐ No

V13_1 - In how many and in which classrooms do you have adjustable tables or chairs?

V14 - To what extent do you think it is important that school furniture is adjustable

according to the body dimensions of students?
Mark with the slider on the bottom line.

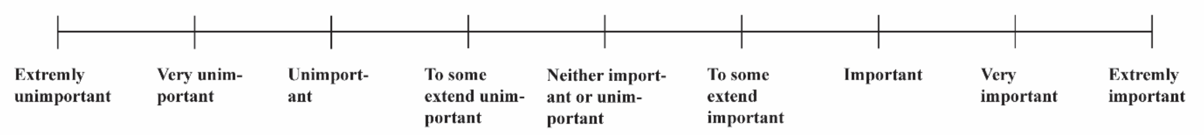

V15 - To what extent would you be prepared to encourage students to use adjustable furniture correctly and regularly (eg height-adjustable chair) during lessons?

Mark with the slider on the bottom line.

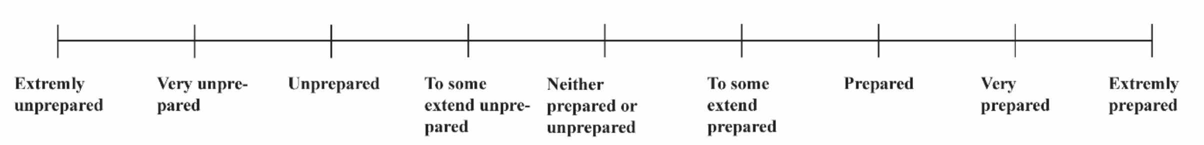

V16 - Do you have school desks in the classrooms that allow you to work (write, read) standing (see the picture for example)?

☐ Yes, in all classrooms
☐ Yes, at least in half of the classrooms
☐ Yes, at least in one classroom
☐ No

An example of a high table with an accompanying chair.
Source: https://www.varidesk.com/shop/by/category/classroom-desks

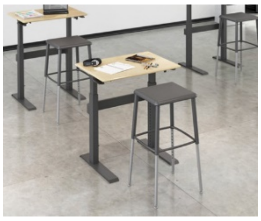

V18 - Imagine having desks at school that allow standing work. Do you think that interrupting prolonged sitting (more than 30 minutes) with standing for 10-15 minutes is feasible during class?

○ Yes
○ No

V18_1 - Why do you think the use of standing desks is not feasible during class?

Example: because of the confusion in the class, because it is not usual.

V19 - To what extent would you be willing to encourage students to stand and use a standing desk with a high chair during lessons?

Mark with the slider on the bottom line.

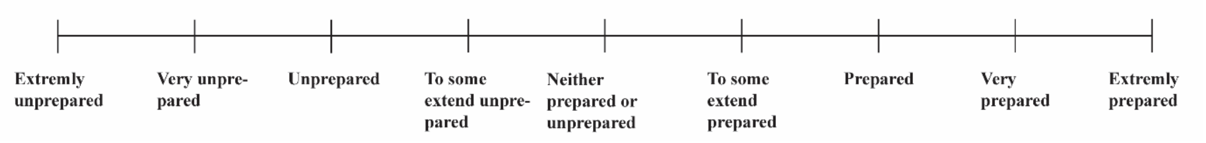

V20 – Do you think that taking active breaks (eg stretching the neck and shoulder muscles, circling the arms, lasting up to 3 minutes) during lessons is feasible?

◯ Yes
◯ No

V21 - Why do you think that taking active breaks is not feasible during class?
Example: because of the confusion in the class, because of the time constraint, because this is not usual.

V22 - Do you take active breaks (eg neck and shoulder stretching, arm rotation, duration up to 3 minutes) during class?

◯ Yes
◯ No

V23 – Do you encourage students to get up during breaks?

◯ Yes
◯ No

V24 - Did you talk about school furniture at any of the pedagogical conferences /meetings/congresses?

◯ No, never
◯ Sometimes
◯ Yes, always

V25 - Have you ever been involved in choosing/ ordering new school furniture?

◯ No, never
◯ Sometimes
◯ Yes, always

V26 - For easier description of the data, we ask for the following demographic data.

V27 - The year of your birth:

Sex:

- ◯ Male
- ◯ Female

V28 - At which institution do you teach?

- ☐ Primary school
- ☐ Secondary school
- ☐ Tertiary school
- ☐ Other:

V29 - Total number of teaching years:

V30 - Number of years of teaching at the current institution:

V31 - Which subject(s) are you teaching??

V32 - How old are the students you teach?

- ☐ 6-10
- ☐ 10-15
- ☐ 15-20
- ☐ 20 or more

V33 - If you wish, please write down the name of the educational institution in which you teach.
